# Incidence of *Clostridium difficile* infection and enteric pathogens in children with inflammatory bowel disease presenting with disease exacerbation

**DOI:** 10.3389/fped.2026.1808589

**Published:** 2026-05-13

**Authors:** Kaltham Al-Shaibah, Reham Jasem, Ali Alsarhan, Christos Tzivinikos

**Affiliations:** 1 Pediatric Department, Al Jalila Children's Hospital, Dubai Health, Dubai, United Arab Emirates; 2 Graduate Medical Education, Mohammed Bin Rashid University of Medicine and Health Sciences, Dubai Health, Dubai, United Arab Emirates; 3 Pediatric Department, Alqassimi Women & Children's Hospital, Sharjah, United Arab Emirates; 4 Pediatric Gastroenterology Department, Al Jalila Children's Hospital, Dubai Health, Dubai, United Arab Emirates; 5 Mohammed Bin Rashid University of Medicine and Health Sciences, Dubai Health, Dubai, United Arab Emirates

**Keywords:** clostridial infection, gastrointestinal microbiota, infectious colitis, inflammatory bowel disease exacerbation, pediatric IBD

## Abstract

**Objective:**

Pediatric patients with inflammatory bowel disease (IBD) are at increased risk of gastrointestinal infections including *Clostridioides difficile* infection (CDI), which can trigger disease exacerbations. Since treatment for disease flares and infections differs, identifying the infection and causative microorganisms is crucial. There is limited data on the frequency of CDI in pediatric patients with IBD in our region. This study aimed to determine the incidence of CDI and other enteric infections in pediatric patients with IBD presenting with symptoms suggestive of a flare, and to identify potential predictors of CDI.

**Methods:**

We conducted a retrospective study from 2017 to 2024, including pediatric patients with inflammatory bowel disease who presented with exacerbation of gastrointestinal symptoms suggestive of a flare-up and underwent stool testing. Demographic and clinical data, including IBD type, treatment regimens, and hospitalization status, were collected. Stool samples were analyzed for infectious pathogens. We analyzed the association between categorical variables using the Chi-square test to identify predictors of *Clostridioides difficile* infection (CDI). Statistical significance was set at a *P*-value <0.05.

**Results:**

Stool testing was performed in 144 encounters, of which 47 (32.6%) were positive for infectious pathogens. The most commonly identified organisms were *Clostridioides difficile* (11.8%) and *E.coli* (11.8%). Patients with Crohn's disease (CrD) had a higher rate of *Clostridioides difficile* infection (CDI) compared to those with ulcerative colitis (UC) (17.2% vs. 8.5%). Patients receiving biological therapies were at significantly higher risk of developing CDI compared to those not on biological therapy (17.9% vs. 6.9%, *P* = 0.042). Among patients hospitalized for flare-up symptoms, one-third were confirmed to have CDI.

**Conclusion:**

Overall, stool testing was positive in over 30% of cases, CDI and *E. coli* were the most common identified infections in pediatric patients with IBD presenting with exacerbating symptoms, highlighting the significance of routine pathogen testing, in patients presenting with symptoms of suggestive of flare. CDI was particularly more prevalent among patients with Crohn's disease, those receiving biological therapy, and those hospitalized for their symptoms. Additionally, our findings highlighted the limited utility of traditional stool culture in detecting enteric infections.

## Introduction

Inflammatory bowel disease (IBD) is a systemic inflammatory disease affecting the gastrointestinal tract. Several factors are known to precipitate IBD exacerbations, including non-adherence to medication regimens, the use of antibiotics and non-steroidal anti-inflammatory drugs (NSAIDs), tobacco use, and gastrointestinal infections (1, 2). *C. difficile* is the most significant contributing agent among the diverse pathogens associated with IBD exacerbation in adult patients (3, 4).

*C. difficile* is a gram-positive, spore-forming, toxigenic bacteria causing a spectrum of gastrointestinal illnesses, ranging from mild diarrhea and abdominal discomfort to severe pseudomembranous colitis, toxic megacolon, and even death (3). Several risk factors have been identified that increase the likelihood of developing *Clostridioides difficile* infection (CDI) in children without IBD, including prior exposure to antibiotic use, history of hospitalization or prolonged hospitalization, the use of gastric acid suppressants, and other comorbidities such as cancer or solid organ transplant (5, 6). Among hospitalized pediatric patients with IBD, the incidence of CDI is 12 times higher than in the general hospitalized pediatric population (7). This elevated risk in patients with IBD is attributed to factors such as decreased microbial diversity and the use of immunosuppressive and antimicrobial therapies (8). Additionally, anti-Tumor Necrosis Factor (TNF) agents have been linked to a greater risk of CDI in adult patients (9). Fecal microbiota transplantation (FMT) has emerged as a potential intervention to restore the disrupted gut microbiome, and it serves as an established treatment for recurrent CDI (10).

It is challenging to distinguish clinically between worsening diarrhea due to IBD exacerbation or intestinal infection, and the two conditions are managed differently. Infections may require antibiotics, while the flare-up may necessitate an immunosuppressant, which can be detrimental during an acute infection (11, 12). Thus, the detection of infections is crucial in patients with IBD presenting with worsening symptoms. The European Crohn's and Colitis Organisation (ECCO) guidance recommends screening for CDI at every disease flare in patients with IBD, particularly in those receiving immunosuppressive therapy (4).

There is a lack of pediatric literature on the incidence of gastrointestinal infections associated with IBD exacerbation among pediatric patients in the Gulf region, but separate societal guidance regarding diagnosis and treatment of CDI in children does exist (13). Our study aims to assess the frequency of CDI and other enteric pathogens associated with disease flares in the pediatric population.

## Methods

### Ethics statement

The Dubai Health Research Ethics Board approved the study.

### Study population, variables, and outcomes

We performed a retrospective cross-sectional study using electronic medical records (EMR) of admitted and non-admitted patients at Al Jalila Children's Specialty Hospital, Dubai Health, United Arab Emirates. We retrieved data for all patients who had International Classification of Diseases (ICD) codes K50, K51, and K52 and who underwent stool testing during an exacerbation of symptoms suggestive of flares between October 2017 and May 2024.

Inclusion criteria included patients between the age of 1 to 18 years old, confirmed diagnosis of IBD as per European Society for Paediatric Gastroenterology, Hepatology and Nutrition (ESPGHAN) Porto IBD subtypes, patients with IBD presenting with exacerbation of symptoms, and those who underwent one of the following tests: gastrointestinal pathogen polymerase chain reaction (PCR) panel, *Clostridium difficile* toxin A & B detection assay (enzyme-linked immunosorbent assay, ELISA), and stool culture. The exacerbation of symptoms was identified as pediatric patients with IBD presenting with fever, diarrhea, abdominal pain, or hematochezia.

We excluded patients who were under 1 year old or over 18 years old, had no confirmed diagnosis of IBD, and those who underwent any of the tests without symptoms suggestive of exacerbation.

We recorded the following values from the medical records: patient demographics, characteristics of IBD disease, presenting symptoms, laboratory testing results, exposure to IBD medication before testing (including whether patients were currently on therapy or had been exposed within the last three months), and treatment therapy.

### Enteric pathogen testing

The gastrointestinal pathogen panel PCR test included 21 analytes: *Campylobacter*, *Clostridium difficile* (toxin A/B), *Plesiomonas shigelloides*, *Salmonella*, *Yersinia enterocolitica*, *Vibrio cholerae*, Adenovirus F40/41, Astrovirus, Norovirus GI/GII, Rotavirus A, Sapovirus, Enteroaggregative *E. coli* (EAEC), Enteropathogenic *E. coli* (EPEC), Enterotoxigenic *E. coli* (ETEC), Shiga-like toxin-producing *E. coli* (STEC), *Shigella*/Enteroinvasive *E. coli* (EIEC), *Cryptosporidium*, *Cyclospora cayetanensis*, *Entamoeba histolytica*, and *Giardia lamblia*. *C. difficile* toxin testing (ELISA) detects toxin A and toxin B.

### Statistical analysis

The primary outcome was to assess the incidence of CDI in pediatric patients with IBD presenting with symptoms suggestive of a flare and identifying common pathogens besides CDI. Secondary outcomes included comparing the rate of CDI in Ulcerative Colitis (UC) vs. Crohn's disease (CrD) patients, assessing the risk of CDI in stricturing and fistulating IBD, measuring calprotectin levels in CDI and comparing them with those without CDI, evaluating the association between various IBD medication exposures and increased risk of CDI, and examining antibiotics used in the treatment of flare.

Age is presented as a median ± standard deviation; categorical variables are described as count and percent. The association between categorical variables and the test results was tested using the Chi-square test. All tests were considered significant with a *P*-value <0.05. The Statistical Package for the Social Sciences (SPSS) 29 software platform was used for statistical analysis.

## Results

### Patient characteristics and clinical presentation

The cohort included 144 encounters and 52 pediatric patients with IBD, of whom 28 (53.8%) had CrD, 19 (36.5%) had UC, and 5 (9.6%) had unclassified IBD (IBDU). The study population consisted of 27 females (51.9%) and 25 males (48.1%), with a median age of 11 years at the time of presentation with exacerbation symptoms and at the time of testing for intestinal infections. Age distribution analysis showed that 38.5% of the participants were adolescents aged between 12 and 18 years, and 42.3% were school-aged children between 6 and 11 years. Preschoolers and toddlers were less common, accounting for 15.4% and 3.8%, respectively. United Arab Emirates (UAE) nationals represented the majority, with 25 participants (48.1%), while the remaining 27 participants (51.9%) were from 14 different countries (Table 1).

**Table 1:** Demographic and disease characteristics of pediatric patients with inflammatory bowel disease presenting with disease exacerbation who underwent stool testing (*n* = 52).

Characteristic	*n* (%)
IBD Subtype	
Crohn's Disease	28 (53.8%)
Ulcerative Colitis	19 (36.5%)
Unclassified IBD (U-IBD)	5 (9.6%)
Gender	
Female	25 (48.1%)
Male	27 (51.9%)
Age Distribution	
Adolescents (12 to 18 years)	20 (38.5%)
School-aged (6 to 11 years)	22 (42.3%)
Preschool (3 to 5 years)	8 (15.4%)
Toddlers (1 to 2 years)	2 (3.8%)
Nationality	
UAE Nationals	25 (48.1%)
Non-UAE Nationals (Bahrain, Oman, Lebanon, Jordan, Palestine, Syria, Iraq, Egypt, Iran, India, Pakistan, France, Dominica, and the United Kingdom)	27 (51.9%)

Clinically, the most frequently reported presenting symptom was abdominal pain, affecting 59.0% of patient encounters. Additional flare symptoms included watery diarrhea (45.8%), hematochezia (41.7%), and fever (28.7%). Calprotectin levels were measured in 67 encounters, with 56 (39.2%) showing elevated levels and 11 (7.7%) having normal values. In the remaining 76 encounters (53.1%), calprotectin levels were not measured (Table 2).

**Table 2:** Clinical presentation fecal calprotectin findings in pediatric inflammatory bowel disease encounters during disease exacerbation (*n* = 144).

Exacerbation Symptoms	*n* (%)
Abdominal Pain	85 (59.0)
Watery Diarrhea	66 (45.8)
Hematochezia	60 (41.7)
Fever	41 (28.7)
Calprotectin Levels	
Elevated Levels	56 (39.2)
Normal Levels	11 (7.7)
Not Tested	76 (53.1)

Stool testing, including stool PCR testing, *Clostridioides difficile* toxin A&B detection assay, and stool culture, was positive in a total of 47 encounters (32.6%). The PCR test alone was positive in 45 encounters (31.3%).

### Positive testing results of *Clostridioides difficile* and predictors of *Clostridioides difficile* infection

Among 144 encounters of pediatric patients with IBD, 17 (11.8%) developed CDI. Of these, 15 cases were identified by PCR and 4 by ELISA, with two of the ELISA-positive cases already detected by the gastrointestinal panel, while stool culture yielded no positive results. There was no statistically significant association between positive *C. difficile* results and various factors, including gender, nationality, presence of penetrating or fistulating disease, elevated calprotectin level, or exposure to IBD medications such as Azathioprine, Methotrexate, Mesalazine, Sulfasalazine, or corticosteroid therapy.

A comparative analysis showed that patients with Crohn's disease had a higher CDI rate compared to ulcerative colitis patients (17.2% vs. 8.5%, respectively), although the difference did not attain statistical significance (*P* = 0.151).

Out of the total encounters included in our cohort, 8 were identified with stricturing disease, and none developed CDI (0.0%). In contrast, among the 58 encounters without stricturing disease, CDI was observed in 11 (19.0%). Regarding fistulating disease, of the 12 encounters identified, 2 developed CDI (16.7%). Among the 54 encounters without fistulating disease, CDI was present in 9 (16.7%).

Examining the incidence of CDI among patients on various IBD medications showed that 62 encounters were on Azathioprine, with 6 developing CDI (9.7%). Conversely, among the 81 encounters not receiving Azathioprine, 10 had CDI (12.3%). Additionally, among 31 encounters on steroids, 3 developed CDI (9.7%). Of the 112 encounters not on steroids, 13 had CDI (11.6%). Remarkably, patients receiving biological therapies had significantly higher rates of CDI (17.9%) compared to those not receiving biological treatment (6.9%) (*P* = 0.042) (Table 3).

**Table 3:** Risk factors and clinical outcomes associated with *Clostridioides difficile* infection in pediatric inflammatory bowel disease encounters.

Risk factor among patient with CDI	CDI positive (*n* = 17), No. (%)	*p*-value
Gender		
Male (*n* = 63)	10 (15.9)	0.182
Female (*n* = 81)	7 (8.6)	
Nationality		
Non-UAE nationals (*n* = 66)	9 (13.6)	0.531
UAE nationals (*n* = 78)	8 (10.3)	
Type of IBD		
Crohn's Disease (*n* = 64)	11 (17.2)	0.151
Ulcerative Colitis (*n* = 59)	5 (8.5)	
Stricturing (In CrD)		
Without stricturing (*n* = 58)	11 (19.0)	0.177
With stricturing (*n* = 8)	0 (0)	
Penetrating/Fistulating Disease (In CrD)		
Without fistulating (*n* = 54)	9 (16.7)	1.000
With fistulating (*n* = 12)	2 (16.7)	
Elevated Calprotectin		
High (*n* = 56)	5 (8.9)	0.086
Recent exposure to IBD Medications (last 3 months)		
Azathioprine (*n* = 62)	6 (9.7)	0.616
Methotrexate (*n* = 1)	0 (0)	0.722
Mesalazine (*n* = 27)	3 (11.1)	0.344
Sulfasalazine (*n* = 31)	2 (6.5)	0.344
Corticosteroid (*n* = 31)	3 (9.7)	0.763
Biologics (*n* = 56)	10 (17.9)	**0**.**042**
Hospitalization Requirement		
Required Hospitalization (*n* = 17)	6 (35.3)	0.700
Treatment Failure		
Lack of symptoms resolution (*n* = 17)	4 (23.5)	**0**.**004**

Bold as its a significant *p* value.

### Clinical outcomes and treatment

The likelihood of hospital admission was similar between patients with and without CDI, showing no significant difference (35.3% and 40.2%, respectively). In terms of clinical response to treatment, lack of symptom resolution was significantly more frequent among patients with CDI compared to those without CDI (23.5% vs. 4.7%). Lack of symptom resolution was defined as an inadequate clinical response within 2 weeks despite the use of the appropriate antibiotics for bacterial infections, excluding the use of supportive care and IBD-specific medications (Table 3). Of the 17 CDI-positive encounters, 12 were treated with metronidazole, 4 were treated with vancomycin, and 1 had missing data.

### Gastrointestinal pathogen PCR panel, *C. difficile* toxin testing (ELISA), and stool culture results

**Figure 1:**
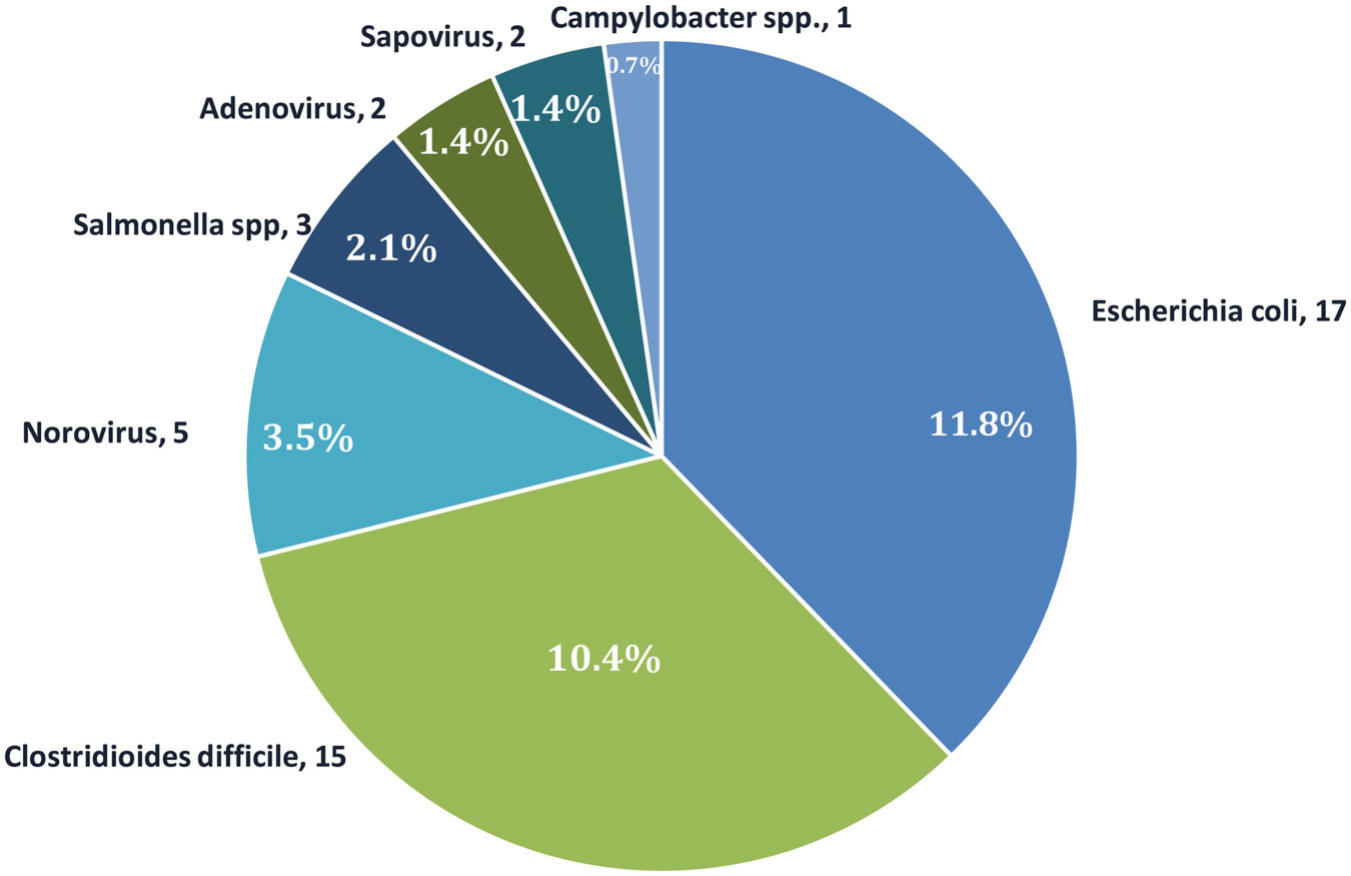
Distribution of enteric pathogens detected by gastrointestinal PCR panel among positive encounters.

The gastrointestinal pathogen panel PCR test was performed in 89 (61.8%) out of 144 encounters, of which it was positive in 45 (31.3%) patient encounters. To reflect overall clinical burden, pathogen frequencies were presented as a proportion of the total number of encounters (144). The most commonly detected pathogens, *E. coli* (11.8%) and *C. difficile* (10.4%), followed by norovirus (3.5%), *Salmonella spp.* (2.1%), adenovirus (1.4%), sapovirus (1.4%), and *Campylobacter spp.* (0.7%) (Figure 1).

The *Clostridium difficile* toxin test (ELISA) results indicated that 4 encounters (2.8%) tested positive, 31 (21.5%) tested negative, and 109 (75.7%) did not undergo testing. Stool culture results showed that 1 encounter (0.7%) tested positive for *Salmonella*, 97 (67.4%) tested negative, and 46 (31.9%) did not undergo stool culture testing (Table 4).

**Table 4:** *Clostridium* toxin test (ELISA) and stool culture test results.

Test type	Number identified (*n* = 144), *n* (%)	Results
*Clostridium* Toxin Test (ELISA)		
Positive	4 (2.8%)	
Negative	31 (21.5%)	
Test Not Performed	109 (75.7%)	
Stool Culture		
Positive	1 (0.7%)	Salmonella
Negative	97 (67.4%)	
Test Not Performed	46 (31.9%)	

## Discussion

In this cross-sectional retrospective single-center study of pediatric patients with IBD presenting with exacerbation symptoms over an 8-year period, gastrointestinal infections were detected in 32.6% of the encounters, with CDI and *E. coli* species being the most common. To our knowledge, this is the first study to evaluate the frequency of *Clostridioides difficile* infection and other enteric pathogens contributing to flares in the pediatric population in the Gulf region, as well as the utility of comprehensive stool testing in pediatric patients with a relapse of IBD. Our findings confirm that pediatric patients with IBD are at high risk of developing CDI and that enteric infections are our findings confirm that pediatric patients with IBD are at high risk of developing CDI and that enteric infections are commonly identified during IBD symptom exacerbations.

In our study, out of 144 encounters, *C. difficile* and *E. coli* infections were each identified in 17 children, making them the most frequently reported pathogens among patients experiencing a disease flare. Our CDI findings are consistent with observations from similar studies conducted in Italy and Canada (14, 15). These results also align with trends documented in the adult IBD population (7, 16). The remaining pathogens were encountered less frequently (Figure 1).

We found no statistically significant association between positive results and gender, nationality, presence of penetrating or fistulating disease, or elevated calprotectin level. However, the analysis revealed that CrD patients exhibited a higher CDI rate compared to those with UC (17.2% vs. 8.5%, respectively). Studies in similar pediatric populations did not report any discernible difference in CDI rates between Crohn's disease and ulcerative colitis patients (7, 14, 15). In contrast, research in adult IBD populations has found higher rates of CDI in UC patients compared to Crohn's disease (7, 16). Potential explanations for this discrepant finding, such as differences in biologic exposure, underlying disease severity, disease location, and patterns of healthcare utilization, have not been explored in previous IBD cohorts and were beyond the scope of the present analysis. These factors, together with the limited sample size of our study, may partially account for the higher CDI rates observed in patients with Crohn's disease.

In our cohort, patients receiving biological therapy demonstrated greater susceptibility to CDI compared to those who were not (17.9% vs. 6.9%). The majority of our patients were on infliximab; these findings were similarly reported in various studies (6, 9, 17). Consistent with our results, most studies exploring the relationship between IBD medications such as 5-Aminosalicylic Acid (5-ASA), corticosteroids, or immunomodulators and the development of CDI have not identified any significant association (4, 15, 18).

Multiple studies have shown worse outcomes for patients with CDI, including higher hospitalization rates and prolonged hospital stays (4). However, in our study, hospitalization rates were similar between patients with and without CDI (35.3% and 40.2%, respectively). Although CDI is generally more common in the elderly, the overall prevalence of CDI in both adult and pediatric hospitalized patients with inflammatory bowel disease showed no significant difference (7).

The majority of our patients with CDI were treated with metronidazole or metronidazole-based regimens, with only a few receiving vancomycin; this was similarly reported in other studies (14). The current consensus for the management of an initial episode of CDI with mild or moderate disease suggests initiating treatment with either oral vancomycin or metronidazole, yet some experts prefer to administer vancomycin over metronidazole for initial treatment. Using the same regimen for the first episode of the first recurrence is recommended. Oral vancomycin must be initiated for a second or any subsequent recurrence and in case of severe disease (19).

Accurate detection of enteric pathogens is crucial for managing flares of IBD, and the PCR panel is widely used. Traditional methods like stool culture have long been used; however, in our study, its effectiveness in detecting enteric infection was highly questioned. Stool culture was performed in 98 encounters, yet it tested positive in only one case (0.7%) of *Salmonella*, which had already been detected by the PCR panel. The use of stool culture as a diagnostic tool for gastrointestinal infections should be reconsidered in the presence of a more sensitive and specific tool that can provide comprehensive microbial testing in patients with IBD (19).

Current diagnostic methods, including PCR and toxin-based assays, have limited ability to distinguish *C. difficile* colonization from true infection in pediatric patients, particularly those with IBD. ECCO guidelines define CDI as the presence of diarrheal symptoms with documented toxigenic *C. difficile* in stool and recommend a two-step diagnostic algorithm, using an initial highly sensitive test (GDH antigen or nucleic acid amplification testing) followed by a confirmatory toxin A/B enzyme immunoassay, to reduce diagnostic misclassification (4).

## Conclusion

Overall, stool testing was positive in more than 30% of cases, with *C. difficile* and *E. coli* the most frequently identified intestinal infections associated with IBD flares, each detected in 11.8% of encounters across all testing methods, highlighting the significance of routine pathogen testing in patients presenting with symptoms suggestive of flare. CDI was particularly more prevalent among patients with Crohn's disease, those receiving biological therapy, and those hospitalized for their symptoms. Additionally, our findings highlighted the limited utility of traditional stool culture in detecting enteric infections, given its low positivity rate (0.7%) and lack of added diagnostic value beyond PCR.

## Limitations

Our findings are limited by the retrospective collection of data and the relatively small number of patients with IBD with *Clostridioides difficile* infection, which may have reduced the statistical power of the analysis. Another limitation is that the study was conducted at a single tertiary care hospital in the UAE, which may not reflect the incidence of CDI in pediatric patients with IBD in other regions or healthcare settings. Although our institution provides care to all patients regardless of nationality, UAE nationals receive treatment free of charge, whereas expatriate patients require insurance authorization or self-payment. As a result, this governmental institution sees a disproportionately higher number of local patients compared with the overall population, which may have introduced selection bias and limited the generalizability of the findings. Additionally, inconsistent application of the recommended two-step *C. difficile* diagnostic algorithm, variations, or incomplete stool testing may have influenced the detection rates of enteric pathogens.

## Data Availability

The raw data supporting the conclusions of this article will be made available by the authors, without undue reservation.
