# Correction: Incidence of *Clostridium difficile* infection and enteric pathogens in children with inflammatory bowel disease presenting with disease exacerbation

**DOI:** 10.3389/fped.2026.1882704

**Published:** 2026-06-08

**Authors:** Kaltham Al-Shaibah, Reham Jasem, Ali Alsarhan, Christos Tzivinikos

**Affiliations:** 1 Pediatric Department, Al Jalila Children’s Hospital, Dubai Health, Dubai, United Arab Emirates; 2 Graduate Medical Education, Mohammed Bin Rashid University of Medicine and Health Sciences, Dubai Health, Dubai, United Arab Emirates; 3 Pediatric Department, Alqassimi Women & Children’s Hospital, Sharjah, United Arab Emirates; 4 Pediatric Gastroenterology Department, Al Jalila Children’s Hospital, Dubai Health, Dubai, United Arab Emirates; 5 Mohammed Bin Rashid University of Medicine and Health Sciences, Dubai Health, Dubai, United Arab Emirates

**Keywords:** clostridial infection, gastrointestinal microbiota, infectious colitis, inflammatory bowel disease exacerbation, pediatric IBD

Author affiliation

The affiliation “Graduate Medical Education, Mohammed Bin Rashid University of Medicine and Health Sciences, Dubai Health, Dubai, United Arab Emirates” was omitted for author “Kaltham Al-Shaibah”. This affiliation has now been added.

The affiliation “Mohammed Bin Rashid University of Medicine and Health Sciences, Dubai Health, Dubai, United Arab Emirates” was omitted for author “Christos Tzivinikos”. This affiliation has now been added.

The original article has been updated.

